# *In-vivo* and *in-vitro* environments affect the storage and release of energy in tendons

**DOI:** 10.3389/fphys.2024.1443675

**Published:** 2024-08-01

**Authors:** Fransiska M. Bossuyt, Timothy R. Leonard, W. Michael Scott, William R. Taylor, Walter Herzog

**Affiliations:** ^1^ Human performance Lab, Faculty of Kinesiology, University of Calgary, Calgary, AB, Canada; ^2^ Laboratory for Movement Biomechanics, Institute for Biomechanics, Department of Health Sciences and Technology, ETH Zurich, Zurich, Switzerland; ^3^ Faculty of Veterinary Medicine, University of Calgary, Calgary, AB, Canada

**Keywords:** Achilles tendon, hysteresis, mechanical properties, musculoskeletal biomechanics, sheep model, experimental -animal models

## Abstract

Understanding tendon mechanical properties, such as stiffness and hysteresis, can provide insights into injury mechanisms. This research addresses the inconsistency of previously reported *in-vivo* and *in-vitro* tendon hysteresis properties. Although limited, our preliminary findings suggest that *in-vivo* hystereses (Mean ± SD; 55% ± 9%) are greater than *in-vitro* hystereses (14% ± 1%) when directly comparing the same tendon for the same loading conditions in a sheep model *in-vivo versus* within 24 h *post-mortem*. Overall, it therefore appears that the tendon mechanical properties are affected by the testing environment, possibly related to differences in muscle-tendon interactions and fluid flow experienced *in-vivo versus in-vitro*. This communication advocates for more detailed investigations into the mechanisms resulting in the reported differences in tendon behaviour. Overall, such knowledge contributes to our understanding of tendon function towards improving modelling and clinical interventions, bridging the gap between *in-vivo* and *in-vitro* observations and enhancing the translational relevance of biomechanical studies.

## Introduction

Tendons are passive structures that consist of clustered collagen fibers connecting muscles to bones. However, the tendons of, e.g., the gastrocnemius and rotator cuff, are common sites of chronic tendon degeneration often leading to tendon rupture associated with pain, disfunction, and disability (Williams et al., 2001; Snedeker and Foolen, 2017). To understand the biomechanics of the musculoskeletal system, tendons have been simplified and represented as spring-like elastic structures, mechanically and anatomically in series with the muscle, hence playing a fundamental role in transferring forces from muscles to bones. Although traditionally, tendons are believed to play a critical role in storage and energy release (Alexander and Bennet-Clark, 1977; Griffiths, 1989), there has been a growing interest in the complexity of the structure of tendons and their function in addition to the storage and release of energy (Benjamin et al., 2008). Researchers in mechanobiology are trying to understand how mechanical loading impacts the biological signalling of tendon cells, but the detailed mechanisms and optimal amount of loading required for injury prevention, treatment, and rehabilitation, remain unknown (Snedeker and Foolen, 2017).

One of the greatest challenges in musculoskeletal biomechanics has been to quantify and correlate tendon strains with the corresponding directly measured *in-vivo* muscle forces to understand how muscles and tendons interact *in-vivo*. Here, both muscle forces and tendon strains are needed to calculate tendon hysteresis (the energy loss during stretching and recoiling of tendons), which is central for understanding the efficiency of everyday movements (Voigt et al., 1995; Adam et al., 2023). Importantly, due to technical limitations and the extremely invasive nature of obtaining *in-vivo* muscle forces and tendon strains, tendon parameters determined *in-vitro* are often used as a substitute to enable an understanding of the *in-vivo* situation. However, tendon hysteresis values obtained *in-vitro* are typically much smaller and more consistent than those obtained *in-vivo* (Finni et al., 2013). This observation raises the question of whether the observed differences in hysteresis properties are actually differences in the mechanical properties of tendons after extraction from the body, or if they rather reflect measurement errors due to the employed technologies.

The aims of this commentary are 1) to provide an overview of *in-vivo* and *in-vitro* obtained hysteresis values in the literature to date as well as 2) to provide direct comparisons of *in-vivo* and *in-vitro* hysteresis values directly measured in the same tendon and for matched loading conditions. We hypothesized that the change of environment impacts the tendon properties and therefore the hystereses obtained in the sheep medial gastrocnemius (MG) tendon *in-vivo* are greater than those measured *in-vitro*.

## Materials and methods

### Literature review

The literature search for this study was conducted in an unsystematic manner. We began by reviewing references cited in a previous publication summarizing the relevant literature on hysteresis obtained in both *in-vivo* and *in-vitro* environments (Finni et al., 2013). To extend the search, the terms “hysteresis,” “tendon,” “force,” “strain” “*in-vivo*,” and “*in-vitro*” were used in PubMed, Google Scholar, and Web of Science. This approach, while comprehensive, did not follow structured protocols and relied on the judgement of the authors for relevance, which may introduce selection bias.

### Experimental procedures

Six healthy female sheep, purchased from a commercial producer, were trained to walk on a motor-driven treadmill at different speeds ranging from a slow walking to slow trotting. Training lasted between 15 and 36 weeks at a frequency of 3–4 sessions per week for 10–15 min per sheep and session. Veterinary care was provided by the University of Calgary Animal Care Unit veterinary team. The Animal Care Committee of the University of Calgary approved all procedures (protocol AC19-0098). Reporting adhered to the ARRIVE (Animal Research: Reporting *In Vivo* Experiments) guidelines (Kilkenny et al., 2010).

### Measurement and surgical procedures

The medial gastrocnemius tendon was isolated and surgically instrumented. Details of the animal care and surgical procedures have been published previously (Bossuyt et al., 2023) and are included in the Supplementary Material S1. A brief description of the surgical procedure is provided here: for the force measurements, a custom made “E”-shaped buckle-type force transducer was attached as an in-series element to the distal portion of the MG (Walmsley et al., 1978; Herzog et al., 1993). For the tendon strain measurements, Sonomicrometry crystals (diameter = 2 mm) with a spatial resolution of 0.016 mm were attached to a section of the MG tendon with a soft surface material (Dragen Skin, stiffness of 1–2 MPa) (Griffiths, 1991; Caputi et al., 1992). The exact placement of the crystals was confirmed post-mortem. All signals were transmitted by telemetry to a custom-built amplifier and synchronized with the use of an electronic synchronization pulse.

### *In-vivo* data collection

The *in-vivo* data were collected 2–3 days following surgery. The sheep walked on the treadmill at different speeds from a slow walking pace to a trot (0.67 m/s, 0.89 m/s, 1.96 m/s) and at different surface inclinations (0°, 1.5°, 3°, 6°) for all speeds. Muscle forces were collected at 1,040 Hz using the buckle-type transducer. Tendon lengths/strains were collected at 520 Hz.

### *In-vitro* data collection

The MG muscle tendon unit, including part of the calcaneus, was dissected for the *in-vitro* experiments which took place 24 h *post-mortem* and included calibration of the tendon force transducer. The tendon was clamped in a mechanical testing machine (Instron, 10 kN load cell, E10000, Norwood, MA, United States) and loaded with 14.9N tare load. After tendon setup, a 20-min rest period was followed by 101 conditioning cycles to 1% strain at 0.5 Hz, and 51 test cycles applying strain rates and peak forces that had been measured during the different walking trials on the previous day. The tendon was kept hydrated throughout the testing using a 0.9% saline solution. Detailed information of the *in-vitro* experimentation procedures is included in the Supplementary Material S2.

### Data analysis

Force and Sonomicrometry data were smoothed using a 4^th^ order recursive Butterworth low-pass filter (cutoff 10 and 50 Hz respectively) (Bossuyt et al., 2023). Data that exceeded unrealistic strain rates (exceeding 74%/s and following visual confirmation), were removed and filled using spline-based interpolations (3^rd^ order cubic spline). The reference length for tendon measurement (length_ref_vivo_) was considered to be the shortest distance between Sonomicrometry crystals observed throughout the trials, allowing *in-vivo* tendon strains across this portion to be calculated as (length-length_ref_vivo_)/length_ref_vivo_)*100. *In-vitro* tendon strains, on the other hand, were measured for the entire tendon length (where length_ref_vitro_ = tendon length at zero strain). Hysteresis was calculated as the difference between the mechanical work stored and the mechanical work released by the tendon during a loading cycle, normalized to the work stored, calculated as the area under the respective force-strain curves.

Finally, to assess level of agreement with previous studies, the data obtained from our *in-vivo* and *in-vitro* analyses were compared against the range of data presented in the literature.

## Results

### Literature confirmation of discrepancy between *in-vivo* and *in-vitro* values

**FIGURE 1:**
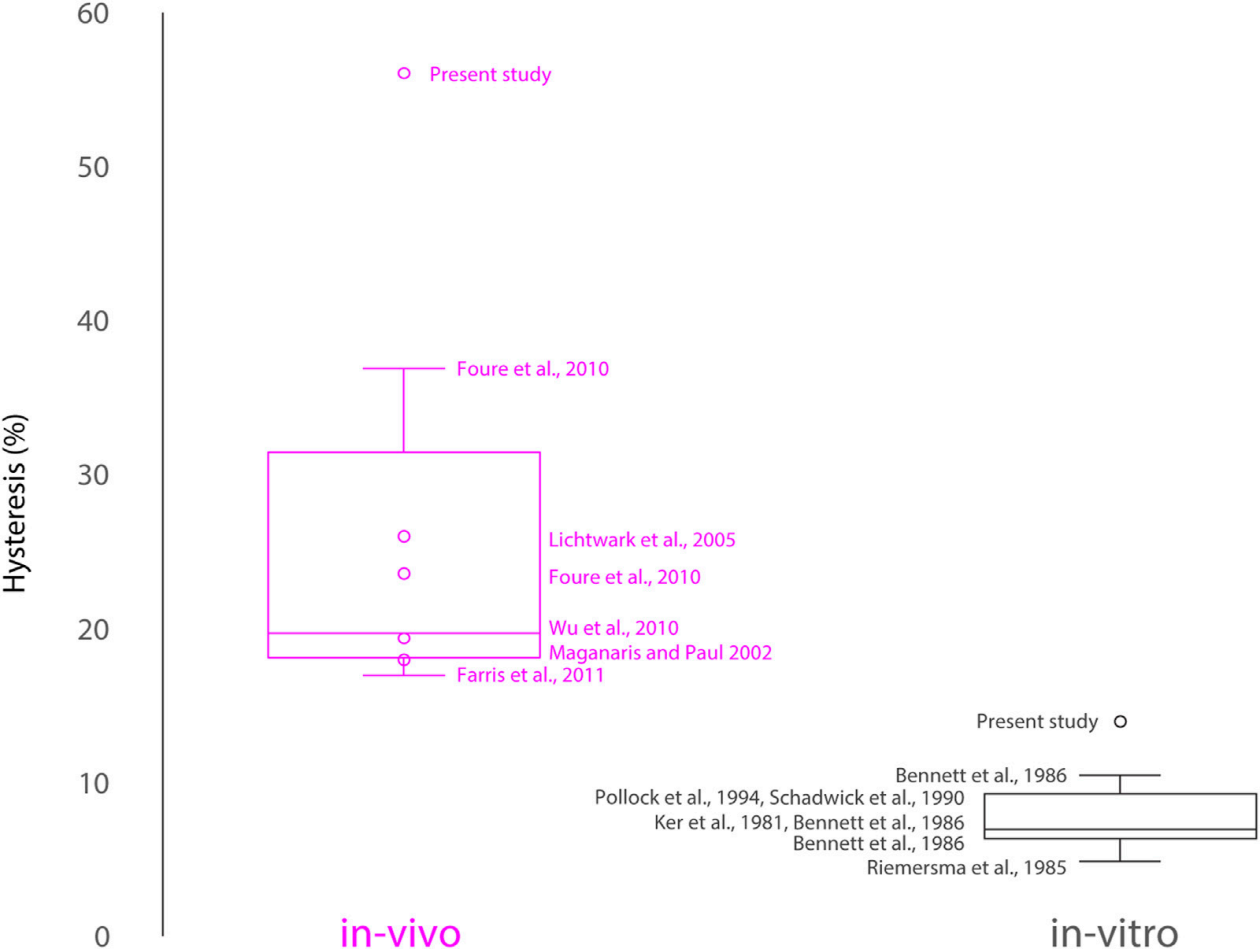
Overview of previously reported hysteresis values of free tendon human and animal *in-vivo* and *in-vitro* experiments. Presented experimental data within this study are the outliers for both *in-vivo* and *in-vitro* data. Note that results of the present study are the first to achieve synchronized measurement of both force and strain data *in-vivo*, but also directly compare the same tissue under comparable mechanical conditions *in-vitro*.

A systematic overview of *in-vivo* and *in-vitro* obtained hysteresis values from human or mammalian gastrocnemius tendon reported in the literature is presented (Figure 1). Here, human Achilles tendon and gastrocnemius tendon specifically were studied *in-vivo* to obtain hysteresis properties before and after plyometric training, as well as during different activities such as isometric plantarflexion contractions, hopping, and treadmill running (Maganaris and Paul, 2002; Lichtwark and Wilson, 2005; Foure et al., 2010; Farris et al., 2011). The latter studies estimated tendon forces from the applied torque and the respective moment arms, and estimated tendon strain using ultrasonography. *In-vitro,* mechanical properties of the plantaris tendon and digital flexor tendons were obtained in several mammals (e.g., sheep, wallaby, deer, and equine), especially towards the end of the 20th century (Ker, 1981; Riemersma and Schamhardt, 1985; Bennett et al., 1986; Shadwick, 1990; Pollock and Shadwick, 1994; Wu et al., 2010). Here, clamping the respective tendons in a mechanical testing machine allow the applied forces and respective strains to be measured directly. Finally, we also included results of the present study for comparison with the published experimental results (Figure 1).

### *In-vivo* and *in-vitro* hysteresis values in the same tendon and for matched loading conditions

**FIGURE 2:**
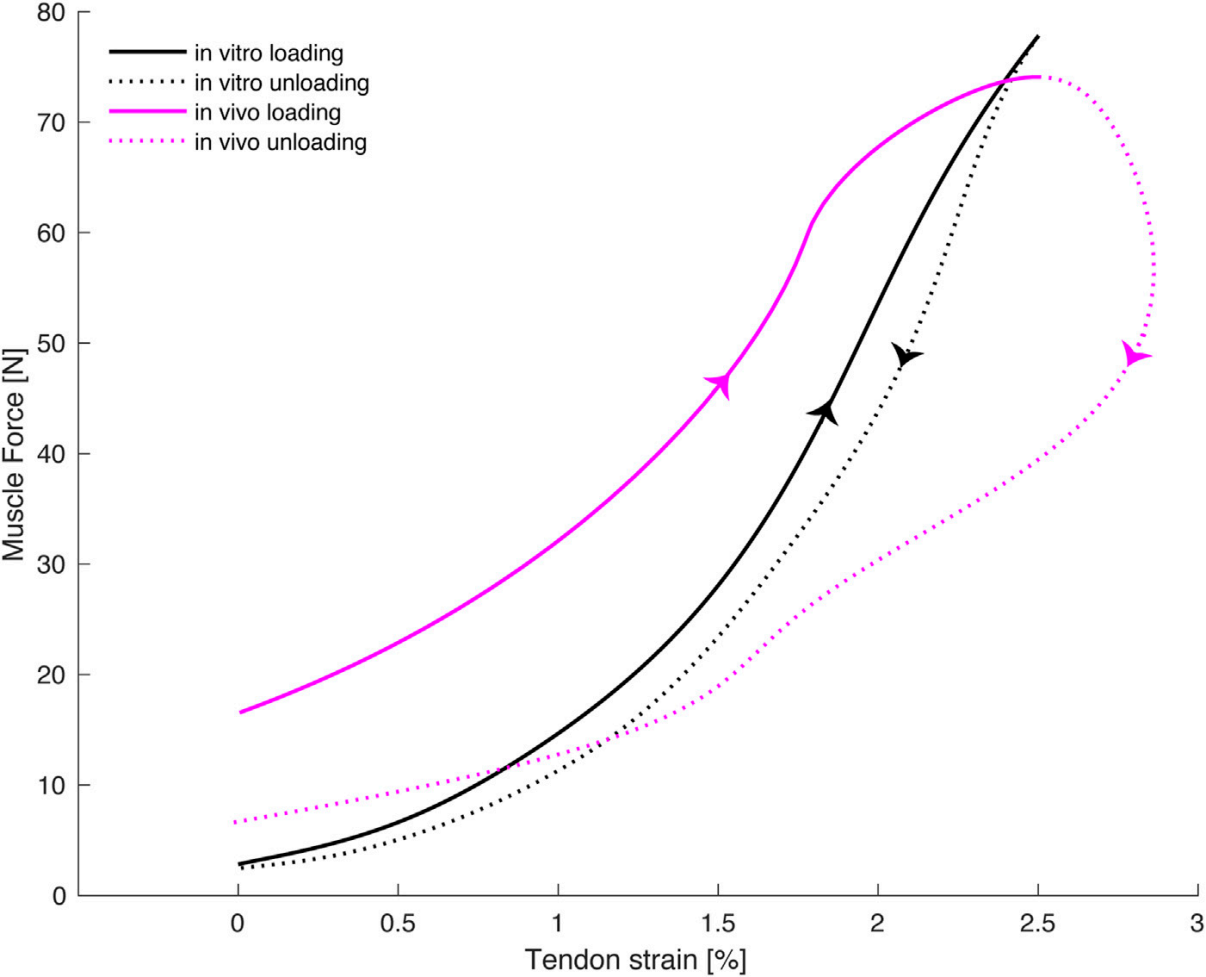
Exemplary tendon strain vs. tendon force obtained for comparable force-time histories under *in-vivo* (purple) and *in-vitro* (black) conditions. Note that the slack length for the *in-vivo* (shortest length measured during all trials) and *in-vitro* conditions (length at zero force, just prior to the onset of force application) were defined differently, and likely caused a shift in tendon strains between the two conditions. To control for this shift, *in-vivo* peak strains were aligned with *in-vitro* peak strains causing a shift of the *in-vivo* hysteresis curves to the left.

Force-strain curves of 3 sequential steps with our sheep walking at 1.96 m/s were captured and used in this analysis (exemplarily shown in Figure 2). The MG tendon hysteresis for the *in-vivo* conditions (Mean ± SD; 55% ± 9%) considerably exceeded those obtained *in-vitro* (14% ± 1%).

## Discussion

The aim of this study was to address the controversy in the literature with regards to tendon hysteresis values obtained *in-vivo versus in-vitro*. Our initial work in presenting a systematic overview of values reported in the literature has confirmed this discrepancy between *in-vivo* and *in-vitro* conditions. These results therefore strongly indicate that the mechanical properties of tendon differ between *in-vivo* and *in-vitro* environments, and that the full complexity of the *in-vivo* environment is likely not yet completely understood. Our own results not only concur with these differences, but further suggest that the differences in hysteresis values are even greater than those reported in the literature.

Typically, average tendon hysteresis values in humans do not exceed 40%, but they have been shown to vary greatly between loading cycles (e.g., 2%–45%) (Farris et al., 2011; Finni et al., 2013). It is important to note that previous work has often been conducted in single laboratories using the same experimental approach. Also, studies focussing on the mechanical properties of aponeuroses *in-vivo*, that have been discussed frequently in the same manner as free tendon properties (Finni et al., 2013), have not been compared within our review (Figure 1). While forces can be readily measured in tendons and are constant along the tendon, the variable and location dependent *in-vivo* aponeurosis forces have eluded measurement and accurate theoretical prediction, thus rendering published mechanical properties of aponeuroses suspect at best, and wrong at worst (Epstein et al., 2006; Herzog, 2019; Bossuyt et al., 2023). Importantly, tendon and aponeuroses hystereses should be studied independently and not be confused interdependently. There are only few studies in which the tendon force-strain hysteresis curves are presented, further limiting comparisons between findings, thus leaving open the question of whether differences between studies are due to methodological differences, the differences in the analyses, or actual differences between tendon properties measured *in-vivo* and *in-vitro.*

Our own investigations show *in-vitro* results that align in magnitude with previous *in-vitro* studies, but the amount of hysteresis obtained *in-vivo* far exceeded previous estimates from *in-vivo* studies involving human tendons (Figure 1). It is important to note that all *in-vivo* data reported until now has determined the tissue level strains but only been able to estimate the actual forces that occur at the tendon. Our study is the first to achieve synchronized measurement of both force and strain data *in-vivo*, but also directly compare the same tissue under comparable mechanical conditions *in-vitro.* The observed differences in mechanical properties in the present study suggests that current *in-vitro* testing methods are not sufficiently able to replicate the conditions occurring *in-vivo.* Possible factors contributing to these differences could include the absence of muscle contraction, the different fluid dynamics, temperature, osmolarity of the environment, edge-effects caused by tendon clamping *in-vitro*, tissue degradation following harvest including potential cell death, and differences in the tissues surrounding the muscle tendon units *in-vivo* and *in-vitro* that may affect the biological responses to loading. Finally, difficulties in defining the *in-vivo* tendon slack length, the differences in boundary conditions between the *in-vitro* (clamping at the ends of the tendon) and *in-vivo* conditions (fixed at bone and muscle/myotendinous junction), and the difference between measuring total vs. local strains for the *in-vivo* and *in-vitro* conditions, might all have affected the mechanical properties of the tendons measured in this study.

In an effort to study the effect of osmolarity on tendon properties during cyclic loading, Buckley et al. (2013) used bathing solutions of different salt concentrations, thereby altering the swelling conditions of tendons. They demonstrated that increased fluid-inflow and internal swelling under hypotonic conditions opposes the lateral contraction of the collagen network required for the tendon to stretch along its longitudinal axis. Although the hysteresis values were not quantified in their study, stress was shown to increase under hypotonic compared to isotonic conditions thereby supporting how osmolarity may impact tendon mechanical properties and add to the complexity of the *in-vivo* environment. To this effect, changes in volume and fluid-outflow during microscopic load transfer between tendon fibrils under stretch have been demonstrated and evaluated using theoretical models (Ahmadzadeh et al., 2015). Furthermore, a recently proposed conceptual framework highlights the role of intratendinous pressure associated with fluid-flow and possible associated mechanisms impacting tendon properties and the pathogenesis of tendon pathology (Pringels et al., 2023). The presented findings and discussions are first steps towards deepening our understanding of the complex *in-vivo* muscle-tendon interaction and demonstrate the importance of considering *in-vivo* fluid-flow and intratendinous pressure when identifying tendon mechanical properties.

There are several limitations in this study. Measuring tendon strains using sonomicrometry is challenging, and many trials of this study could not be used for analysis because of the noisy tendon strain data. *In-vitro* pilot testing using sonomicrometry gave excellent results that could not be reliably obtained during *in-vivo* testing. We are not certain as to the differences in sonomicrometry measurements during *in-vivo* and *in-vitro* testing but the challenges encountered during *in-vivo* testing might be related to the difficulty in securely attaching the sonomicrometry crystals to the tendinous tissue *in-vivo*, the encapsulation of the crystals during the period between surgical implementation and *in-vivo* testing, possible dislocation of the crystals by neighbouring tissues, the inability to adjust the sonomicrometry parameters properly after implantation and recovery, signal artifacts introduced by the skeletal tissues in the vicinity of the tendon, and artifacts produced by tendon fluid exchange. Also, *in-vivo* tendon strains were obtained for part of the tendon, for which the location could not be accurately reproduced between tendons, while *in-vitro* tendon strains were measured for the entire tendon using the Instron materials testing machine. Since non-uniformities in local tendon strains have been reported (e.g., Franz et al., 2015; Maas et al., 2020; Adam et al., 2023), the local strains measured in our study *in-vivo* might not be representative of the total tendon strains measured *in-vitro*. Finally, our *in-vitro* experiments did not control for the temperature changes that have been observed previously in-vivo during a sustained gallop in horses (Wilson and Goodship 1994). Despite these limitations, our findings support the idea that tendon properties, particularly hysteresis obtained *in-vivo* and *in-vitro* differ for the same tendon and similar loading patterns.

## Conclusion

We conclude that mechanical properties of tendons in general, and hystereses of tendons specifically, differ when obtained under *in-vivo* or *in-vitro* conditions. If confirmed in further studies, these results would have important implications for understanding fundamental biomechanics and injury mechanisms, as well as musculoskeletal models that are based on *in-vitro* properties of tendons and possibly other soft tissues, such as ligaments, menisci, and aponeuroses.

## Data Availability

The raw data supporting the conclusions of this article will be made available by the authors, without undue reservation.
