# Supplementary material for: In-vivo and in-vitro environments affect the storage and release of energy in tendons
Source: Front Physiol. 2024 Aug 1;15:1443675. doi: 10.3389/fphys.2024.1443675 (PMC11324601; doi:10.3389/fphys.2024.1443675)
Supplement: Supplementary file 1 [file DataSheet2.pdf]

Supplementary material S2

Detailed information on in-vitro experimentation procedures

A custom designed stainless-steel clamp with a dowel (diameter 10mm) shape and rough surface replicating sandpaper were used to clamp the tendon for the in-vitro experiments (Figure 1). The part of the calcaneus connected to the tendon was clamped with a stainless-steel rectangular clamp (Figure 2). These clamps were selected following pilot-tests and allow the tendon to wrap around the dowel while providing pressure at the back surface. Following placement of the specimen (Figure 3), both clamps were tightened with 7 Nm and the tendon was lengthened to zero-strain (defined by increasing the tendon length until there is a change in baseline force drift of 0.5N). The tendon was kept hydrated throughout testing using a 0.9% saline solution.

Prior to experimental testing the tendon length, width, and thickness were measured at zero-strain using a digital calliper. Width and thickness were measured at the proximal end of the tendon, middle section, as well as at the distal end. Three measurements were taken at each location (i.e., 3x7 measurements) by a single examiner while a second examiner made notes. The average of the three assessments were used for further calculations (Table 1).
Next, the tendon was lengthened until tare load was reached. Tare load was defined based on pilot tests and calculated by multiplying 0.272 Pa x the cross-sectional area (54.73 mm^2) of the tendon. More specifically, the force preload was selected to ensure the forces are greater that the 4N of noise in the 10kN load cell (+/- 2N). Tendon length at pre-load was measured similarly as described above (Table 1).

$$Tare\ load = 0.272\ Pa\ x\ (pi\ x \left(\frac{tendon\ width}{2}\right) x \left(\frac{tendon\ thickness}{2}\right))$$

A 20-minutes rest period was provided while the tendon remained in the pre-load position followed by 101 warm-up cycles at 1% strain cyclic loading (1.03 mm). The rest period of 20-minutes was provided following every loading test preventing damage to the specimen due to overloading and fatigue. The experimental condition that replicated the in-vivo loading of walking at 1.97 m/s included 51 test cycles where the tendon was stretched up to 2.75% (2.82 mm) with a strain rate for the loading period of 28.2 mm/s, for the unloading of 14.8 mm/s, followed by 0.19s of rest/swing in between.

Following experimental testing, the tendon was weighted using a digital laboratory scale (7.13g).

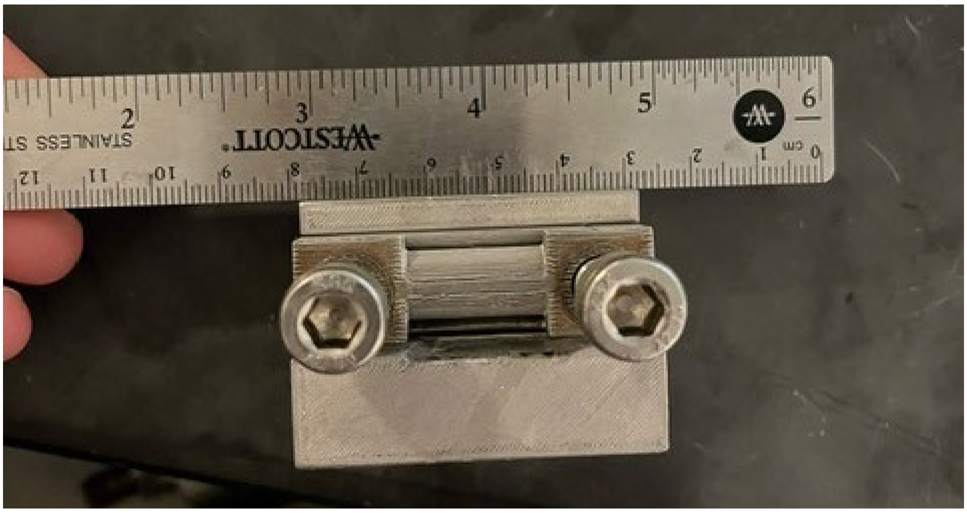

Figure 1: A custom designed stainless-steel clamp with a dowel (diameter 10mm) shape and rough surface replicating sandpaper were used to clamp the tendon for the in-vitro experiments.

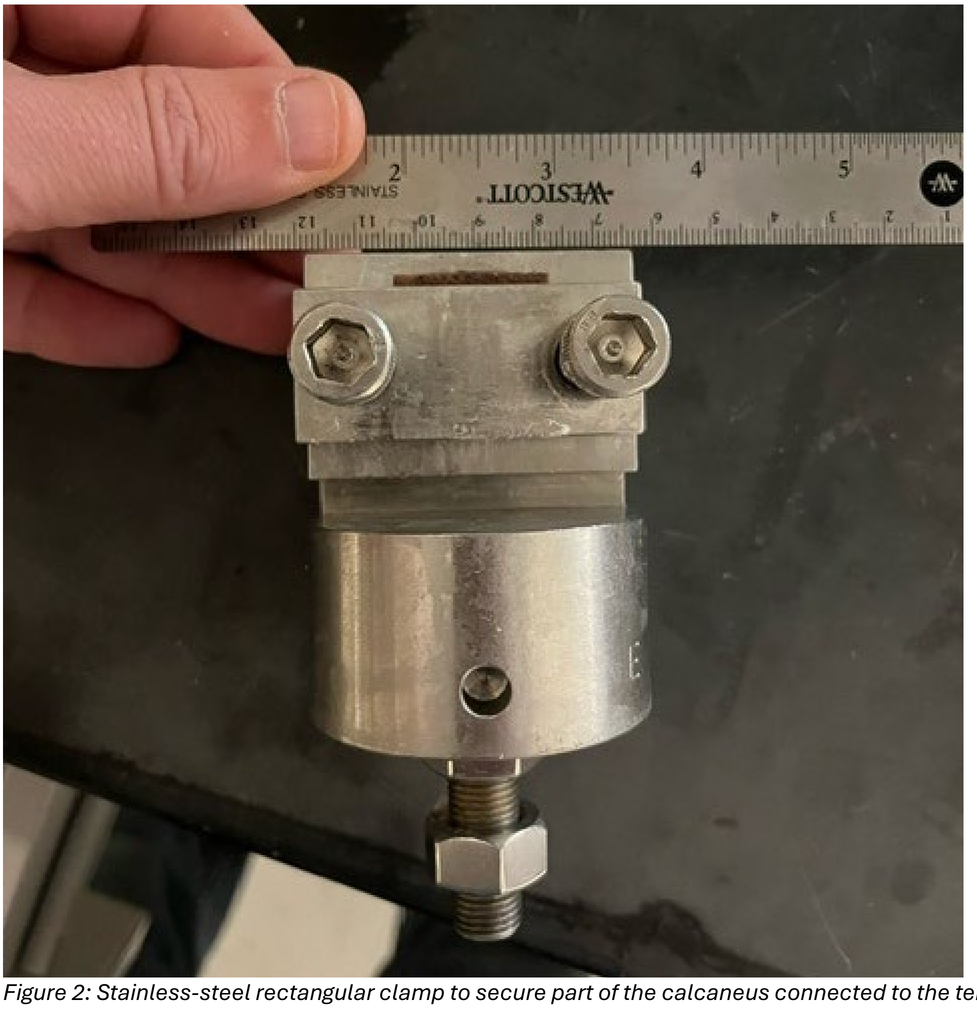

Figure 2: Stainless-steel rectangular clamp to secure part of the calcaneus connected to the tendon.

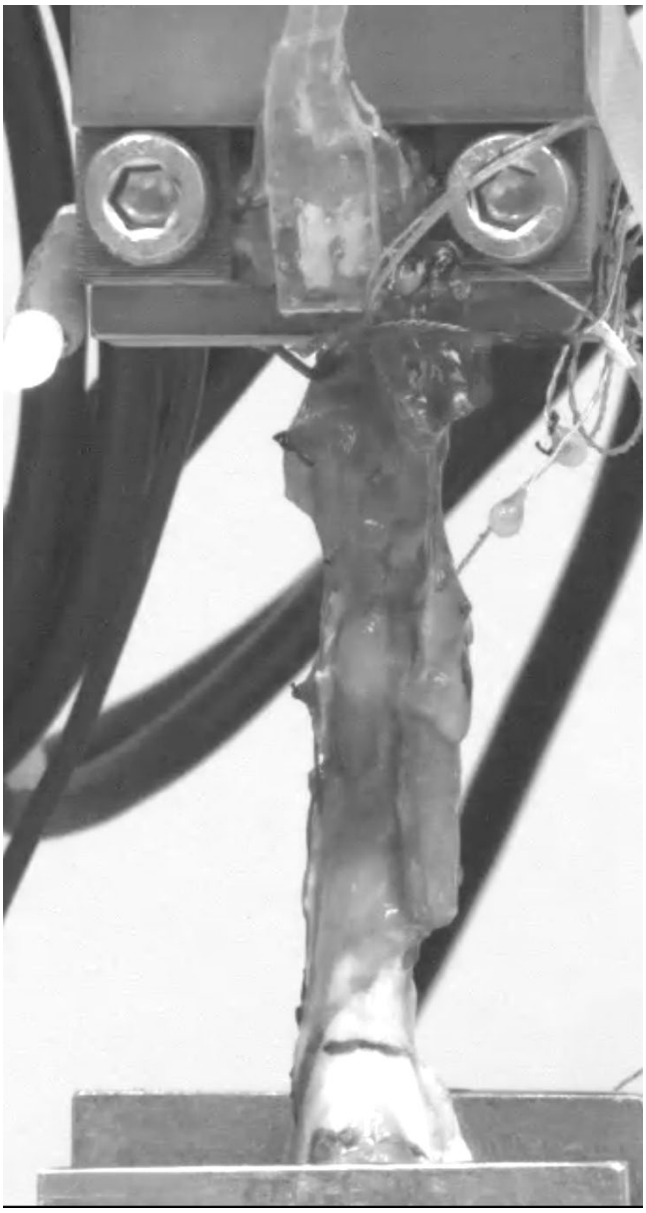

Figure 3: Medial gastrocnemius clamped in the mechanical testing machine.

Table 1: Measurement assessments of sheep medial gastrocnemius tendon in mechanical testing machine (Instron). All measurements are in mm and were taken with a digital calliper by a single examiner.

		**Length (mm)**	**Width (mm)**				**Thickness (mm)**			
			Prox	Mid	Dist	Average	Prox	Mid	Dist	Average
**Zero-strain**	Value 1	98.3	9	9.4	13		6.5	7.1	7.9	
	Value 2	99.2	9	9.3	13		6.6	6.8	7	
	Value 3	99.9	8.7	9.2	12.7		5	7	6.6	
	Average	**99.1**	8.9	9.3	12.9	**10.4**	6.03	6.97	7.17	**6.7**
**Pre-load**	Value 1	102.5								
	Value 2	102.6								
	Value 3	102.4								
	Average	**102.5**								
