# Supplementary material for: In-vivo and in-vitro environments affect the storage and release of energy in tendons
Source: Front Physiol. 2024 Aug 1;15:1443675. doi: 10.3389/fphys.2024.1443675 (PMC11324601; doi:10.3389/fphys.2024.1443675)
Supplement: Supplementary file 2 [file DataSheet1.pdf]

Supplementary material S1

A sample of six healthy female sheep (breed: Suffolk-cross and purebred Île de France, age: 17±3 months, weight: 40.3±4.9 kg). Female sheep were selected because of the smaller size and ease of handling, it was decided not to include rams to avoid the risk of injury and of pregnancy across sheep. All sheep were purchased from a commercial producer and trained (10–15 min sessions per animal, 3–4 times per week, for 15–33 weeks) to walk and trot on a motor-driven treadmill at different speeds ranging from a slow walking pace to a trot (0.67 m/s, 0.89 m/s, 1.34 m/ s, 1.96 m/s) at different inclines (0◦, 1.5◦, 3◦, 6◦). Sheep were housed in a small group and individual pens with open rail design that allowed for visual and nose to nose contact at the University's veterinary campus. Diet included measured volumes of hay, commercial sheep supplement, and loose mineral and ad lib water. Access to an outdoor grassed paddock was provided when the weather conditions allowed. Husbandry was provided by the facility animal care team with animals checked twice daily. Veterinary care was overseen by the University of Calgary Animal Care Unit veterinary team.

Sheep were fasted 15 hours prior to surgery. The sheep were sedated before general anaesthesia (Dexdomitor, 0.015 mg/kg and Alfaxalone, 2mg/kg: intravenous) on the surgery day. Vital signs including heart rate, core temperature, oxygen saturation and breathing rate were monitored throughout surgery as sheep were maintained at ~1-2% isoflurane. The left hindlimb and back were shaved and cleaned using Hibitane© soap and 70 % ethanol. If required, additional sedation was administered (Dexdomitor, 0.004 mg/kg: intravenous). Using aseptic surgery, an incision was made on the lateral side of the left hind limb from the calcaneus to the distal end of the MG. The MG tendon was isolated, and the force transducer was secured on the tendon using a nylon monofilament. Two Sonomicrometry crystals were attached to a soft surface material (Dragon Skin, stiffness of 1-2 MPa) using a 4–0 silk suture onto the MG. The leads of the force transducer and the Sonomicrometry crystals were routed subcutaneously from the hind limb to the lower spine (L1-L4) and soldered to a custom-made connector containing a built-in amplifier (AD627, Analog Devices, Wilmington, Massachusetts, USA). The Sonomicrometry leads were attached to a separate titanium connector. All incisions were closed using 4–0 Vicryl/PDS and 3–0 Ethilon sutures. Thirty minutes prior to the end of the surgery, isoflurane anesthesia was discontinued while medical oxygen was maintained, and analgesic drugs (Meloxicam, 0.5 mg/kg: subcutaneous; Buprenorphine, 0.01 mg/kg: intravenous) were administered. Atipamazole (5x volume of Dexdomitor: intravenous) was used to reverse the anesthesia. Animals were placed in postoperative recovery and monitored closely, receiving analgesics (Buprenorphine, 0.02 mg/kg and Meloxicam 0.5 mg/kg: subcutaneous) every 12 h until the day of data collection. Following the in-vivo data collection, Dexdomitor (0.015 mg/kg: intravenous) was administered after which the animal was injected with an overdose of Sodium Pentobarbital (240 mg/ml at a dose of 100 mg/ kg: intravenous) and euthanized.

BOSSUYT, F. M., ABRAMOVIC, S., LEONARD, T., SAWATSKY, A., SMITH, C. R., TAYLOR, W. R., MICHAEL SCOTT, W. & HERZOG, W. 2023. The non-intuitive, in-vivo behavior of aponeuroses in a unipennate muscle. J Biomech, 147, 111430.
